# Proof by synthesis of *Tobacco mosaic virus*

**DOI:** 10.1186/gb-2014-15-5-r67

**Published:** 2014-04-30

**Authors:** Bret Cooper

**Affiliations:** 1 Soybean Genomics and Improvement Laboratory, USDA-ARS, Beltsville, MD 20705, USA

## Abstract

**Background:**

Synthetic biology is a discipline that includes making life forms artificially from chemicals. Here, a DNA molecule was enzymatically synthesized *in vitro* from DNA templates made from oligonucleotides representing the text of the first *Tobacco mosaic virus* (TMV) sequence elucidated in 1982. No infectious DNA molecule of that seminal reference sequence exists, so the goal was to synthesize it and then build viral chimeras.

**Results:**

RNA was transcribed from synthetic DNA and encapsidated with capsid protein *in vitro* to make synthetic virions. Plants inoculated with the virions did not develop symptoms. When two nucleotide mutations present in the original sequence, but not present in most other TMV sequences in GenBank, were altered to reflect the consensus, the derivative synthetic virions produced classic TMV symptoms. Chimeras were then made by exchanging TMV capsid protein DNA with *Tomato mosaic virus* (ToMV) and *Barley stripe mosaic virus* (BSMV) capsid protein DNA. Virus expressing ToMV capsid protein exhibited altered, ToMV-like symptoms in *Nicotiana sylvestris.* A hybrid ORF6 protein unknown to nature, created by substituting the capsid protein genes in the virus, was found to be a major symptom determinant in *Nicotiana benthamiana*. Virus expressing BSMV capsid protein did not have an extended host range to barley, but did produce novel symptoms in *N. benthamiana*.

**Conclusions:**

This first report of the chemical synthesis and artificial assembly of a plant virus corrects a long-standing error in the TMV reference genome sequence and reveals that unnatural hybrid virus proteins can alter symptoms unexpectedly.

## Background

*Tobacco mosaic virus* (TMV) has been a model system in biology for more than 100 years and has led to the discovery of some of the basic underpinnings of life [1-4]. It has contributed to fundamental biology and to the applied sciences, being used to establish genetic foundations of mutation and plant immunity and to create the first transgenic crop plant with an improved agricultural trait [5-7].

**Figure 1:**
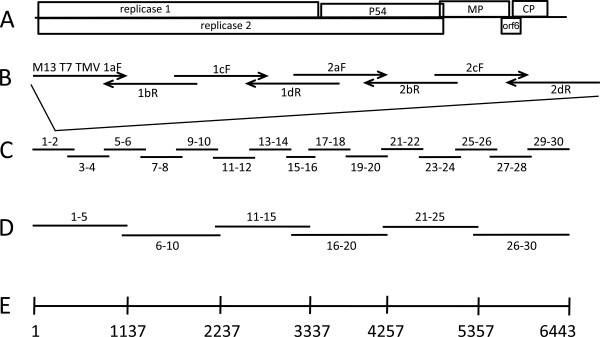
**Schematic of synthetic TMV. (A)** Genome organization. **(B)** Long-oligonucleotides with 20 or 40 base overlaps. **(C)** Overlapping approximately 480 bp molecules made from long-oligonucleotides in (B). **(D)** Overlapping approximately 1,140 bp molecules made from (C). **(E)** Full-length DNA made from molecules from (D).

Two variant RNA sequences of TMV (GenBank V01408.1 and V01409.1) were deduced by Goelet *et al.* in 1982 [8], making TMV the first plant pathogen and one of the first life forms to have its genome resolved. The sequences confirmed that the virus encodes at least four proteins (Figure 1): the 126 kDa replicase component; the 183 kDa replicase component that arises from translational read-though of the amber termination codon of the 126 kDa protein gene; a 30 kDa movement protein (MP) required for TMV RNA translocation between cells; and a 17.5 kDa capsid protein (CP) that enables long distance spread between leaves and stable transmission between plants [9]. A fifth, 54 kDa protein, predicted as the difference between the two replicase components, has an undefined virological role if any [9], and a sixth, 4.8 kDa protein, ORF6, encoded by an open reading frame (ORF) overlapping the MP and CP genes, influences symptomology [10].

The sequence of TMV variant V01408.1, herein dubbed the Goelet sequence, became a reference by which all other plant viruses were compared, and it facilitated the creation of TMV cDNA from which infectious RNA transcripts could be expressed [11], opening new possibilities for characterizing the encoded genes and for transiently expressing foreign genes in plants [12]. Amidst all this success, however, is the almost forgotten fact that the Goelet sequence does not represent a single molecule of a TMV genome. Rather, it is a composite consensus of more than 400 independent clones of cDNA fragments reverse transcribed from a population of TMV RNA molecules exhibiting nucleotide polymorphisms consistent with the inherent error rate of viral RNA-dependent replication. Complicating matters is the likelihood that the virus that Goelet *et al.* sequenced was a mixture of closely related strains [13]. Today, the Goelet sequence is the National Center for Biotechnology Information (NCBI) reference sequence for TMV (NC_001367.1). In 2009, its accession was updated to include the ORF6 protein, but to this date the genomic nucleotide sequence has not been amended. Furthermore, sequences for more than a hundred TMV variants or strains have been reported and none has been merged with NC_001367.1. Consequently, BLAST alignment of NC_001367.1 to all other full-length TMV genomic sequences in the partially non-redundant NCBI Nucleotide collection yields only one hit with perfect identity: itself. Meanwhile, other TMV variants have been molecularly cloned and demonstrated to be infectious [11,14], but there is no known infectious clone for the Goelet sequence, and it remains unknown whether the Goelet sequence represents an infectious viral molecule.

The molecular biology techniques that first enabled the cDNA cloning of TMV 32 years ago have matured to the point where it is now possible to synthesize DNA molecules from basic chemicals in the absence of precursor biological template molecules such as RNA. This advance is part of the domain called synthetic biology, a discipline that can be used to verify the veracity of genome sequences, perform archeology, and assemble genomes *de novo*[15,16]. *Poliovirus* was the first virus to have its genome chemically synthesized based on an alphabetic text blueprint of its nucleotide sequence, a feat later performed for other animal and bacterial viruses [16-25]. The complete chemical synthesis of a plant virus genome, however, has not yet been reported. To achieve this mark, DNA was enzymatically synthesized from overlapping oligonucleotides based on the Goelet alphabetic rendering of TMV and was then transcribed into RNA that was artificially packaged into virions with purified TMV CP *in vitro*. The man-made virus particles encoding the Goelet sequence, a mutant thereof, and two viral chimeras were tested in plants.

## Results and discussion

### Goelet sequence DNA synthesis from modular clones

Thirty sets of long-oligonucleotides with complementary, overlapping ends were chemically synthesized for construction of a linear DNA molecule encoding the Goelet TMV sequence (Figure 1). The DNA molecule also was designed to encode the T7 RNA polymerase promoter for RNA transcription *in vitro* and to have flanking sequences for PCR amplification. Sets of long-oligonucleotides were assembled to create 15, approximately 480 bp DNA modules (Figure 1) that were cloned in plasmids and sequenced. Only six clones for each construct needed to be screened to find at least one with a perfect module sequence, except in one case where it required the screening of 13 clones. The frequency of correct to incorrect modules and the error rate were similar to those previously reported for modules used to synthesize the mouse mitochondrial genome [26].

DNA was amplified from the modules, and the full-length DNA molecule of the Goelet sequence, called 1-30, was enzymatically synthesized from overlapping intermediates using 3 PCR steps for a total of 60 cycles (Figure 1) and was accomplished in 2 to 3 days by the author (fragment size selection by gel purification was the most time consuming step). This time estimate is consistent with the 5-day estimate reported for the PCR synthesis and enzymatic assembly of the mouse mitochondrial genome, which is roughly double the size [26].

7-Methyl guanosine capped-RNA was transcribed *in vitro* from gel purified PCR product of 1-30 and was inoculated to *Nicotiana tabacum* cv. Xanthi *NN* (tobacco), but no hypersensitive local lesions, the classic marks of infection, appeared. By contrast, the same amounts of RNA from DNA made by PCR amplification of the control plasmid pFL-TMV-NA, a known infectious TMV cDNA clone expressed by a T7 promoter [14,27], did produce local lesions on *NN* tobacco. FL-TMV-NA RNA, however, only produced 4 to 10 local lesions per leaf, which suggested low inoculation potential. Because TMV RNA is susceptible to nuclease degradation and is less infectious than packaged virus [28], the transcribed RNA was encapsidated *in vitro* with CP isolated from purified FL-TMV-NA virions. On the basis of the number of local lesions, encapsidation improved the infectivity of control FL-TMV-NA RNA by three to five times (purified CP preparations by themselves did not produce symptoms). The CP then was used to encapsidate 1-30 RNA, but the preparation still did not produce local lesions on *NN* tobacco or mosaic symptoms on Xanthi *nn* tobacco (SX), the classic systemic-symptom host. The experiment was repeated, but again 1-30 virus was not infectious.

### Uncommon polymorphisms in the Goelet sequence

The lack of symptoms implied that 1-30 DNA may not be error-free. To examine its integrity, 1-30 DNA was ligated into a plasmid and four clones were sequenced. Three clones perfectly matched the Goelet sequence whereas the fourth contained a single, silent mutation. Encapsidated RNA transcripts prepared from one plasmid with a perfect Goelet sequence produced no symptoms on *NN* and SX tobacco. Therefore, the integrity of 1-30 DNA was not to blame. Instead, the Goelet sequence itself was suspicious.

**Figure 2:**
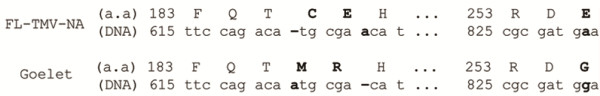
**Sequence polymorphisms between the Goelet sequence and FL-TMV-NA.** Alignment of DNA nucleotides from positions 615 to 632 and 825 to 833. The corresponding translated amino acids (a.a) are shown, and the differences are in boldface type. This region is in the replicase.

The DNA sequence of control pFL-TMV-NA was compared to the text of the Goelet sequence and three major differences were found. pFL-TMV-NA contains substitutions that add restriction endonuclease sites *Nde*I before the MP start codon and *Afl*II before the MP stop codon. These substitutions have no detrimental effects on viral pathogenicity. pFL-TMV-NA also contains a G to A substitution at nucleotide position 832 (with respect to the Goelet sequence), which leads to an E instead of G at amino acid position 255 in the replicase (Figure 2). This is a unique polymorphism that also serves as a distinguishing marker for FL-TMV-NA. The other deviation is an A deletion at nucleotide position 624 followed by an A insertion five bases later that preserves the integrity of the reading frame, but changes two consecutive amino acids in the replicase at positions 186 and 187 from MR to CE (Figure 2). This last deviation was noticed as early as 1990, but no effect was attributed to it [29].

To evaluate the prevalence of the MR polymorphism among TMV isolates, the Goelet amino acid sequence from amino acids 166 to 207 was compared to the NCBI non-redundant protein sequence database by BLASTP. Identical matches were made to the MR amino acids in the first seven reported TMV replicase records, three of which referenced the Goelet sequence. The CE amino acids, however, appeared in 58 of the following TMV replicase records. The next match in the BLASTP report to deviate from CE was that for *Tomato mosaic virus* (ToMV), a closely related tobamovirus, which encoded CR instead. The C at amino acid position 186 is also conserved in other tobamoviruses such as *Bell pepper mottle virus*, *Tobacco mild green mosaic virus*, *Sunn-hemp mosaic virus*, and *Odontoglossum ringspot virus*. Hence, the lack of prevalence of the MR polymorphism among TMV isolates and related tobamoviruses implies that the Goelet DNA sequence for nucleotides 624 to 629 may be an artifact of reverse transcription, DNA sequencing, or human error.

### An infectious, synthetic TMV

**Figure 3:**
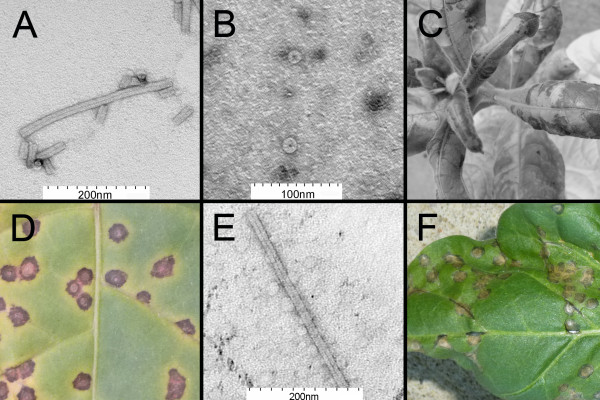
**Encapsidation and infectivity of synthetic 1-34CE-30 and 1-34CE-ToMV CP-30. (A)** 1-34CE-30 RNA encapsidated *in vitro* with FL-TMV-NA CP (stained with uranyl acetate and imaged by transmission electron microscopy; distance between first and last black ticks in white bar = 200 nm). **(B)** FL-TMV-NA CP disks prior to the addition of 1-34CE-30 RNA (stained with uranyl acetate and imaged by transmission electron microscopy; distance between first and last black ticks in white bar = 100 nm). **(C)** Mosaic symptoms on *N. tabacum* cv. Xanthi SX plant inoculated with encapsidated 1-34CE-30 (4 weeks after inoculation). **(D) ***N. tabacum* cv. Xanthi *NN* local lesions from 1-34CE-30 are to the left of the midrib and local lesions from 1-34CE-ToMV CP-30 are to the right. **(E)** 1-34CE-ToMV CP-30 virion from Xanthi SX (stained with uranyl acetate and imaged by transmission electron microscopy; distance between first and last black ticks in white bar = 200 nm). **(F) ***N. sylvestris* infected with 1-34CE-ToMV CP-30.

The MR amino acid polymorphism is suspicious because it is uncommon among TMV replicases and because it arises in the Goelet sequence from an insertion and deletion event rather than the more prevalent single nucleotide substitution. To see if the MR sequence explained why RNA from 1-30 was not infectious, new clones for module 3-4 were made from long-oligonucleotides encoding CE, and linear DNA for 1-34CE-30 was amplified and transcribed. This time, synthetic virions of 1-34CE-30 produced classic mosaic symptoms on SX and local lesions on *NN* tobacco (Figure 3A,C,D). Viral cDNAs amplified from local lesions encoded CE amino acids, but they did not contain the G to A polymorphism at nucleotide position 832. Therefore, the virus causing the symptoms was derived from the synthetic 1-34CE-30 DNA molecule and not FL-TMV-NA virions used to manufacture the CP preparation.

### Capsid protein complementation

It has been known since the 1950s that the CPs from tobamoviruses like *Holmes ribgrass virus* can transencapsidate homologous TMV RNA *in vitro*[30]. At the time of discovery, the results inspired the question whether CP genes were interchangeable among tobamoviruses, but it took the advent of molecular biological techniques to test the idea. In an early report of plant viral genome recombination, an infectious cDNA of ToMV was engineered to express the CP gene of TMV [31]. The feat, however, required the generation of separate cDNAs for ToMV and TMV, restriction endonuclease mapping, subcloning, and site-directed mutagenesis with phage to create a chimera that had additional sequence alterations in the MP gene and the CP promoter [31]. Here, using more precise synthetic techniques, the interchangeability of CPs was revisited. The ORF sequences for the 1-34CE-30 CP gene and the ToMV CP gene were swapped within a text file, and 10 corresponding long-oligonucleotides were synthesized. Full-length DNA was amplified from perfect plasmid clones for two new modules, and RNA was transcribed and encapsidated *in vitro*. The chimera synthetic virus 1-34CE-ToMV CP-30 produced local lesions on Xanthi *NN* within 5 days (Figure 3D) and mosaic symptoms on Xanthi SX within 7 days. The rapid systemic infection in SX indicated that the ToMV CP complemented long-distance transport function in lieu of the TMV CP [32]. Mass spectrometry confirmed the presence of ToMV CP distinguishing peptides in the *NN* local lesions, and this evidence also verified that the symptoms were not a result of contaminating FL-TMV-NA from the CP preparation (Table 1). Transmission electron microscopy confirmed ToMV CP encapsidation of the chimera virus progeny in Xanthi SX (Figure 3E).

**Table 1:** Virus peptides identified from plants by tandem mass spectrometry

**Inoculum**	**Sample**	**Peptide**	**Spectra**	**Missed cleavages**	Ions score*	***P*-value**	**Observed peptide mass**	**Hypothetical peptide mass**	**Charge state**	**Record annotation**
1-34CE-ToMV CP-30	*NN* local lesion 1	RVDDATVAIR	2	1	50	3.20E-03	558.3115	1114.6095	2+	ToMV CP
		IIEVENQQSPTTAETLDATR	2	0	122	3.50E-11	1108.5559	2215.0968	2+	ToMV CP
1-34CE-ToMV CP-30	*NN* local lesion 2	RVDDATVAIR	2	1	50	2.40E-03	558.3115	1114.6095	2+	ToMV CP
		IIEVENQQSPTTAETLDATR	2	0	128	2.10E-11	1108.5561	2215.0968	2+	ToMV CP
1-34CE-ToMVnoorf6-30	*Nicotiana benthamiana*	RVDDATVAIR	3	1	58	1.20E-03	558.3113	1114.6095	2+	ToMV CP
		SAINNLVNELVR	6	0	81	4.30E-06	671.3783	1340.7412	2+	ToMV CP
		IIEVENQQSPTTAETLDATR	1	0	105	2.20E-09	1108.5542	2215.0968	2+	ToMV CP
		TTVQQQFSEVWKPFPQSTVR	1	0	89	1.70E-08	1197.1170	2392.2176	2+	ToMV CP
1-34CE-BSMV CP-30	*N. benthamiana*	SQVAEYLAALDR	3	0	104	4.90E-08	668.3484	1334.6830	2+	BSMV CP
		GQIGLPNYLPAPK	1	0	49	4.50E-04	684.3877	1366.7609	2+	BSMV CP
		ATTNPSPPAQAPSENLTLR	1	0	109	2.00E-09	983.0071	1963.9963	2+	BSMV CP

*Mascot was used for peptide-spectrum matching.

TMV and ToMV can be distinguished by the symptoms they produce on *N. sylvestris* that has the *N’* gene, which confers hypersensitive resistance to ToMV but not to wild-type TMV. *N’* encodes a protein with a coiled-coil domain, nucleotide binding site, and a leucine-rich repeat like many other plant disease resistance genes [33]. The elicitor of *N*’ was mapped indirectly to the ToMV CP gene by Saito *et al.*[31], who replaced the ToMV CP with an homologous TMV CP gene restriction fragment in an infectious ToMV clone and observed the loss of *N’* elicitation. Around the same time, Dawson and colleagues [34,35] showed that some RNA mutations in the TMV CP gene were sufficient for eliciting *N’*, and their data also indirectly implicated the ToMV CP gene in elicitation. It was later affirmed that variants of TMV CP, and not TMV CP gene RNA, indeed elicited *N’*[36,37], but the same has never been shown for ToMV CP, although this is a technicality since the results for mutant TMV CP clearly implicate ToMV CP as well. Nevertheless, it was necessary to examine synthetic chimera 1-34CE-ToMV CP-30 virus on *N. sylvestris* to confirm the hypothesis that it would exhibit a ToMV-like phenotype. So leaf extract from 1-34CE-ToMV CP-30 virus-infected Xanthi SX was inoculated to *N. sylvestris*. Local lesions appeared within 3 days (Figure 3F). By comparison, 1-34CE-30 virus produced systemic symptoms, but no local lesions on *N. sylvestris*. Thus, this experiment directly demonstrates that the *N’* elicitor is included within the coding region of the ToMV CP gene.

**Figure 4:**
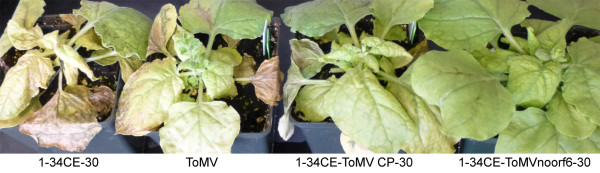
**Effects of ORF6 on symptomology.** Symptoms on *N. benthamiana* 14 days after inoculation with the respective viruses.

**Figure 5:**
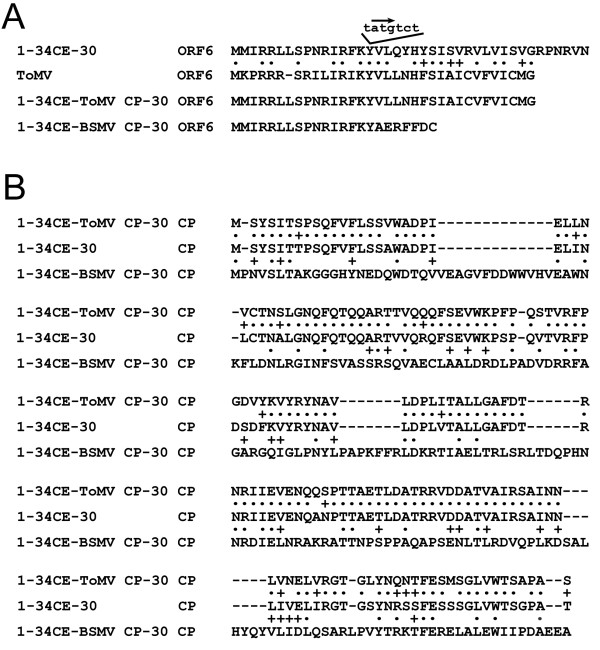
**Needleman-Wunsch amino acid alignment for ORF6 and CP of the respective viruses. (A)** ORF6. The nucleotide position for the CP gene start codon is shown by the arrow. **(B)** CP. Dots indicate identical amino acids; plus signs indicate similar amino acids.

Additional experiments, the descriptions of which will follow, were required to validate the ToMV CP proper as the elicitor, but there were unintended consequences with synthesizing 1-34CE-ToMV CP-30 DNA that first needed to be reconciled. TMV and ToMV also can be distinguished by the symptoms they produce on *Nicotiana benthamiana.* For example, 1-34CE-30 virus caused severe epinasty, chlorosis, curling, and lethal systemic leaf and stem necrosis (Figure 4). Symptoms by ToMV were less severe by comparison, but still detrimental (Figure 4). Symptoms produced by chimera virus 1-34CE-ToMV CP-30 on *N. benthamiana* were more similar to those produced by ToMV (Figure 4). DNA sequencing of reverse transcription-PCR (RT-PCR) products from infected *N. benthamiana* revealed no sequence mutations in 1-34CE-ToMV CP-30. Therefore, these results appeared to implicate the TMV/ToMV CP genes in symptomology in *N. benthamiana*, reminiscent of the way they did in *N. sylvestris*. Confounding a final conclusion, however, was the ORF6 gene, which overlaps the TMV MP and CP genes. Since TMV ORF6 has been implicated in symptomology [10], it was possible that the slightly milder symptoms of ToMV were attributed to ToMV ORF6, which is shorter than and divergent to TMV ORF6, especially in the carboxyl terminus region, which is encoded within the overlapping CP gene (Figure 5A). Similarly, the ToMV-like symptoms of 1-34CE-ToMV CP-30 virus may have been attributed to the hybrid ORF6 arising from replacing the coding region of the TMV CP gene with the coding region of the ToMV CP gene; the final 16 of 34 amino acids of the hybrid ORF6 are derived from ToMV as well. To determine the role of hybrid ORF6 in symptomology, the codons for the first two methionines of ORF6 were mutated to eliminate potential translational starts (the mutations were silent to the MP). Symptoms for the new virus, 1-34CE-ToMVnoorf6-30, were even milder on *N. benthamiana* compared to the other viruses with functional ORF6 genes (Figure 4). DNA sequencing of RT-PCR products confirmed that the ORF6 translational start mutations were retained; no mutations were found in the rest of the viral genome. Mass spectrometry verified the production of ToMV CP (Table 1). Thus, while it is not ruled out that ToMV CP is not a symptom determinant in *N. benthamiana*, it is clear that ORF6 is. The results imply that the carboxyl end of ORF6 that overlaps the TMV CP gene influences symptoms the most. The results also reveal that unnatural hybrid proteins created by viral genome synthesis can unpredictably alter disease symptoms.

Because hybrid ORF6 influences symptoms in *N. benthamiana* and is partly encoded by the ToMV CP gene to which *N’* gene elicitation is mapped, it was necessary to rule out whether hybrid ORF6 contributes to elicitation. Thus, 1-34CE-ToMVnoorf6-30 virus, isolated from *N. benthamiana*, was inoculated to *N. sylvestris*. Local lesions appeared 3 days later. Hence, *N’* elicitation is attributed to the part of the gene that encodes the ToMV CP, not the part that encodes ORF6. Like the type strain of ToMV, 1-34CE-ToMV CP-30 virus does not have a mutation in its ToMV CP gene RNA. Thus, RNA structural alterations encoded by the ToMV CP gene do not explain elicitation of *N’*. Hence, ToMV CP is the elicitor of *N*’.

**Figure 6:**
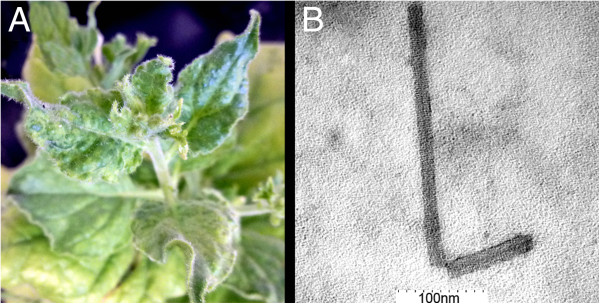
**Synthetic 1-34CE-BSMV CP-30. (A)** Mosaic symptoms on *N. benthamiana*. **(B)** Virion from *N. benthamiana* (stained with uranyl acetate and imaged by transmission electron microscopy; distance between first and last black ticks in white bar = 100 nm).

Finally, CP complementation was explored between TMV and the heterologous *Barley stripe mosaic virus* (BSMV), a rod-shaped virus that exhibits CP sequence divergence to TMV and ToMV (Figure 5B). Although barley, *Hordeum vulgare* cv. Black Hulless, is a natural host for BSMV but not TMV, it was demonstrated that in the presence of a mixed infection with BSMV, TMV RNA transencapsidated by BSMV CP could be recovered from upper leaves of barley [38,39]. It can be hypothesized from these results that the BSMV CP aids the systemic spread of TMV in barley, but the role for BSMV CP in extending the host range of TMV has remained unknown since the 1970s. To examine this, oligonucleotides were designed from the BSMV CP gene text and used to replace the TMV CP gene to create 1-34CE-BSMV CP-30 DNA. This design also resulted in a truncated TMV ORF6 with just seven amino acids provided by the overlapping BSMV CP gene sequence (Figure 5A). The derivative chimera virus did not produce any symptoms on inoculated Xanthi SX leaves, but it did produce TMV-like localized necrosis on the inoculated leaves of *N. benthamiana* 3 weeks after infection. The virus moved systemically 1 week later and produced unique mosaic symptoms without the systemic necrosis and stunting previously seen with 1-34CE-30 or 1-34CE-ToMV CP-30 viruses (Figures 4 and 6A). DNA sequencing of viral genome RT-PCR products from infected *N. benthamiana* revealed a single mutation, T to A at position 6,415 in the 3′ untranslated terminal region. This mutation is uncommon among TMV isolates; it was not present in the template DNA modules and may have arisen from DNA or RNA polymerase error, or it may have evolved in the plant. BSMV CP distinguishing peptides were identified from the infected *N. benthamiana* by mass spectrometry (Table 1), and transmission electron microscopy confirmed BSMV CP encapsidation of the chimera virus (Figure 6B). Together these results imply that the unique symptoms were a result of the BSMV CP or the hybrid ORF6, although the point mutation cannot be discounted. Infected *N. benthamiana* leaves were used as inocula for Xanthi *NN* and barley. Local lesions appeared on Xanthi *NN* within 4 days, but after several attempts at inoculating and then growing barley for 3 weeks at 30°C as described for mixed infections [38], the viral chimera could not be identified by RT-PCR from upper leaves. So, even though this chimera does not explain the spread of TMV in barley in a mixed infection with BSMV [38,39], it does, like the previous TMV/ToMV chimera, reveal that synthetic chimera viruses can produce unexpected and unusual symptoms.

## Conclusions

The number of organisms with sequenced genomes has grown in recent years, yet the veracity of nearly all of these genome sequences remains hypothetical. Synthetic biology provides a means for proving whether a sequence is a true blueprint for life. To date, only a few viral sequences and a lone prokaryotic genomic sequence have been authenticated [17,40]. This cannot be said, however, for the original TMV sequence [8]. RNA transcribed from an enzymatically synthesized DNA template of the Goelet sequence is not infectious. The cause appears to be related to insertion/deletion polymorphisms not in most other TMV sequences. The polymorphisms lead to the translation of MR amino acids instead of CE amino acids at positions 186 and 187 of the 126 kDa and 183 kDa replicase components. It is possible that MR disrupts the replicase methyltransferase domain critical for interactions with host proteins or with the 3′ end of TMV RNA [41-44]. The origins of the deleterious polymorphisms are not known with certainty, but it is unlikely that they were native to the TMV studied by Goelet *et al*. Thus, this report identifies a longstanding error in a model, reference sequence, but also proves that a corrected sequence sustains biological replication (Figure 3C).

Synthetic biology also can be used to identify the functions of genes. The TMV CP gene was exchanged with the homologous ToMV CP gene to test complementation. The chimera virus, 1-34CE-ToMV CP-30, moved systemically through tobacco, and this verified that ToMV CP can complement the long-distance transport function of the TMV CP. Furthermore, the chimera, as expected, produced ToMV-like local lesions rather than TMV-like systemic mosaic in *N. sylvestris* (Figure 3F). The local lesions, controlled by *N’*, were elicited by ToMV CP translated from the inserted gene. This insertion, however, also created a hybrid ORF6 gene overlapping the reading frames of the TMV MP gene and the exchanged ToMV CP gene (Figure 5A). Unexpectedly, the novel, hybrid ORF6 protein contributed to symptom severity in another plant, *N. benthamiana* (Figure 4).

While ToMV CP was sufficient for complementing systemic spread of chimera virus 1-34CE-ToMV CP-30 in three tobacco hosts, BSMV CP was not sufficient for enabling the systemic spread of chimera virus 1-34CE-BSMV CP-30 in barley, a non-host for TMV. Thus, transencapsidation of TMV by BSMV in barley may only be a consequence of mixed infection and may not explain spread in the non-host [39]. It is possible that the BSMV triple-gene-block, which is analogous in function to the TMV MP, is needed to support the spread of TMV in barley [45]. Nevertheless, 1-34CE-BSMV CP-30 virus did spread systemically through *N. benthamiana* and produced unique mosaic symptoms instead of TMV-like systemic necrosis (Figure 6A). It is possible that the novel symptoms were a result of replacing the TMV CP with the BSMV CP, which also led to a potential truncated TMV/BSMV hybrid ORF6 protein (Figure 5A). Thus, it should be clear that the inconspicuous ORF6 and its unnatural derivatives created by synthetic recombination can have pathological consequences. Embedded in the TMV genome are several other small ORFs that have not been examined for pathological importance, possibly because of the past difficulty of using traditional molecular biology techniques to decouple these potential ORFs from the critical replicase, MP, and CP genes in which they are embedded. It should be feasible to use synthetic biology to restructure the ORFs in the TMV genome and separate the genetic responsibilities of these potential, undiscovered genes much in the same way the bacteriophage genome was refactored [20,21].

Synthetic genomes have been previously created for a few animal and bacterial viruses [16-25]. These genomes were usually maintained in DNA plasmids or were circularized for infectivity. By contrast, with the exception of the non-infectious Goelet replica, the synthetic DNA templates of the infectious plant viruses studied here were not maintained in bacterial plasmids. As linear DNA molecule templates, they are benign and incapable of natural biological replication either in bacteria or plants. Because TMV is an RNA virus, synthetic RNA needed to be transcribed *in vitro* from DNA. To improve infectivity, virions were constituted *in vitro* by combining the synthetic RNA with purified TMV CP; virion formation was confirmed by electron microscopy (Figure 3A). These particles may be considered to be the first synthetic virions because they were assembled *in vitro* from purified CP and synthetic RNA transcribed from synthetic DNA in the absence of potential host factors [19], although there is opportunity yet to supersede this achievement by formulating fully synthetic virus components entirely by macromolecular chemistry. That detail is trivial, however, compared to the more poignant observation that these man-made plant viruses with simple mutations and recombined genomes altered symptoms in both expected and unexpected ways, portending the ramifications of the artificial manufacture of inherently stable infectious entities [25]. Consequently, all plants and experimental materials that came into contact with synthetic virions and progeny were appropriately disposed.

## Materials and methods

### Template design

The basic nucleotide blueprint was the M13 (-20) primer sequence as an anchor, the T7 minimal promoter sequence immediately followed by the DNA conversion of the Goelet sequence (GenBank Refseq NC_001367.1; 6395 nucleotides), and a terminal *Kpn*I cleavage sequence (total length 6,443 nucleotides; Figure 1). *Kpn*I cleavage of a plasmid encoding TMV adds five nucleotides to the end of the virus but does not inhibit TMV RNA infectivity [14]. The *Kpn*I cleavage site sequence was added for PCR amplification in light of the predicted high degree of secondary structure in the TMV 3′ end [46]. The CP DNA sequences for ToMV (GenBank X02144.1) and BSMV (GenBank U35772.1) were also used.

### Oligonucleotide design

Long-oligonucleotides with complementary overlapping ends were designed from the blueprint to allow construction of DNA by overlapping PCR [47]. A modular strategy was adopted such that larger DNA molecules could be built from smaller units (Figure 1). Sets of 4 or 6 long-oligonucleotides were designed to overlap each other by 40 bases, with the exception of sets 15 and 16, which overlapped each other by 220 bases, and the first and last, which did not overlap with each other, thereby maintaining the linearity of the molecule (Additional file 1). Long-oligonucleotides were designed regardless of their potential secondary structures. Amplification oligonucleotides (Ampoligos), the lengths of which were optimized for 55 to 65°C annealing temperatures for PCR, anchored the ends of each module. Oligonucleotides were procured from Invitrogen (Carlsbad, CA, USA), ordered on a 25 nmol scale, and diluted to 100 μM in water.

### DNA construction

Each long-oligonucleotide (0.5 μl) of two consecutive sets of long-oligonucleotides (Figure 1) was annealed in 16 to 18 μl TE buffer by heating to 95°C for 1 minute and cooling to 30°C at 0.1°C/s. Annealed long-oligonucleotides (0.5 μl) were reacted with 4.5 μl synthesis reaction mix (1 μl 5× isothermal reaction buffer [48], 0.1 unit Phusion DNA polymerase (Thermo Scientific, Waltham, MA, USA), 16 units *Taq* DNA ligase (New England Biolabs, Ipswich, MA, USA), and water) at 50°C for 1 h. The assembly (0.5 μl) was amplified by PCR for 20 cycles using amplification oligonucleotides and Phusion Hot Start II DNA polymerase (Thermo Fisher Scientific). DNA was separated by agarose-gel electrophoresis. DNA fragments were purified using UltraClean 15 (MO BIO Laboratories, Carlsbad, CA, USA).

### DNA cloning

DNA molecules approximately 480 bp long made from 2 sets of overlapping long-oligonucleotides and amplified in 20 PCR cycles were inserted into pCR-Blunt II-TOPO (Invitrogen) and propagated and selected in *Escherichia coli*. Cloned plasmids were sequenced by Genewiz (Germantown, MD, USA) using the Sanger method. For some 1-30 full-length molecules, DNA was amplified with oligonucleotides with *Xho*I and *Kpn*I restriction endonuclease sites, digested, and ligated into pUC18 digested with *Sal*I and *Kpn*I, and the plasmids were transformed into Stbl2 *E. coli* (Invitrogen).

### RNA transcription

7-Methyl guanosine capped-RNA was produced from 250 to 1,000 ng DNA using the mMESSAGE mMACHINE T7 transcription kit (Ambion/Life Technologies, Carlsbad, CA, USA). Reactions were supplemented with 0.5 μl GTP. A PCR product (using Ampoligos 1 F and 30 R) of plasmid pFL-TMV-NA, a derivative of pU3/12 with unique *Nde*I and *Afl*II restriction sites near the start and stop codon of the MP gene [14,27], was evaluated as a control.

### Viral RNA encapsidation

Virions of FL-TMV-NA and 1-34CE-ToMV CP-30 were purified from infected Xanthi SX, and virions of 1-34CE-BSMV CP-30 were purified from infected *N. benthamiana*[49]. FL-TMV-NA CP was purified from virions using the glacial acetic acid degradation method [50]. RNA (2 μg) was encapsidated in a reaction with 100 μg of purified FL-TMV-NA CP [30].

### RT-PCR

RNA was extracted from 1 cm^2^ leaf disks from plants showing viral symptoms using the RNeasy Plant Mini Kit (Qiagen, Germantown, MD, USA). RNA (10 μl) was annealed to Ampoligo 30 R and reverse transcribed using SuperScript III (Life Technologies, Carlsbad, CA, USA). cDNAs of viral genomes were amplified using Ampoligos and were sequenced by Genewiz using the Sanger method.

### Mass spectrometry

Protein was extracted from 1 cm^2^ leaf disks from plants showing viral symptoms by grinding in liquid nitrogen and precipitating in acetone/10% trichloracetic acid/0.07% beta-mercaptoethanol. Extracts were centrifuged at 21,000 × *g* for 15 minutes and washed two times in acetone. The residue was dried by vacuum centrifugation and resuspended in 100 μl 100 mM Tris base pH 8.5 and 8 M urea. Concentrations of soluble protein were determined by bicinchoninic acid assay (Pierce/Thermo Fisher Scientific, Carlsbad, CA, USA). Soluble protein (300 μg) was reduced, derivatized, digested in trypsin and analyzed by reverse phase-tandem mass spectrometry as previously detailed [51]. Tandem mass spectra were analyzed with Mascot 2.4.0 against virus protein records in the NCBI NR protein sequence database. Peptides with *P*-values less than 0.05 were accepted. Spectra matching viral peptides were confirmed not to have equivalent or better matches to plant protein records in NR. Tandem mass spectrometry data (RAW files) are archived at [52].

## Abbreviations

bp: base pair; BSMV: *Barley stripe mosaic virus*; CP: capsid protein; MP: movement protein; NCBI: National Center for Biotechnology Information; ORF: open reading frame; PCR: polymerase chain reaction; RT-PCR: reverse transcription-PCR; SX: Xanthi *nn* tobacco; TMV: *Tobacco mosaic virus*; ToMV: *Tomato mosaic virus*.

## Competing interests

The author declares that he has no competing interests.

## Authors’ contributions

The author was responsible for experimental conception, design, execution, analysis and interpretation of data; drafting and revising the article; and final approval of the version to be published.

## Supplementary Material

Additional file 1: Table S1 Oligonucleotide sequences used for constructing synthetic viral genomes and the sequences of the assembled DNA constructs of the synthetic viral genomes. Click here for file
